# Pre-Pregnancy Provegetarian Food Pattern and the Risk of Developing Gestational Diabetes Mellitus: The Seguimiento Universidad de Navarra (SUN) Cohort Study

**DOI:** 10.3390/medicina60111881

**Published:** 2024-11-16

**Authors:** Vanessa Bullón-Vela, Ainara Martínez-Tabar, Maddi Etxezarreta-Uranga, Miguel Ángel Martínez-González, Francisco Javier Basterra-Gortari, Maira Bes-Rastrollo

**Affiliations:** 1 Department of Preventive Medicine and Public Health, University of Navarra, 31008 Pamplona, Spain; mbullon@alumni.unav.es (V.B.-V.); amartinez.80@alumni.unav.es (A.M.-T.); metxezarret@alumni.unav.es (M.E.-U.); fj.basterra.gortari@navarra.es (F.J.B.-G.); mbes@unav.es (M.B.-R.); 2 IdiSNA, Navarra Institute for Health Research, Irunlarrea 3, 31008 Pamplona, Spain; 3 CIBER Fisiopatología de La Obesidad y Nutrición, 28029 Madrid, Spain; 4 Department of Nutrition, Harvard T. H. Chan School of Public Health, Boston, MA 02115, USA; 5 Department of Endocrinology and Nutrition, Hospital Universitario de Navarra, Universidad Pública de Navarra, 31008 Pamplona, Spain

**Keywords:** gestational diabetes mellitus, provegetarian food pattern, plant-based diet

## Abstract

*Background and Objectives:* Gestational diabetes mellitus (GDM) is one of the most common medical conditions in pregnancy, with adverse effects on maternal and neonatal outcomes. Evidence suggests a beneficial effect of plant-based dietary patterns, rich in foods derived from plant sources and low in animal foods, on type 2 diabetes; however, their effects on GDM remain unclear. We aimed to investigate the association between pre-pregnancy provegetarian food patterns and the incidence of GDM in a Spanish cohort. *Materials and Methods:* This subsample of the Seguimiento Universidad de Navarra (SUN) cohort analyzed 3589 Spanish university graduate pregnant women with a mean (standard deviation) age of 28 (±4.3) who were initially free of pre-existing diabetes at baseline. Dietary food consumption was evaluated through a validated, 136-item semi-quantitative food frequency questionnaire. The pre-pregnancy provegetarian food pattern was obtained by assigning positive scores to plant-based food groups and reverse scores to animal food groups. Energy-adjusted quintiles were applied to allocate points to construct the provegetarian food pattern, ranging from 12 to 60 points. Logistic regression models were performed to estimate the odds ratios (OR) of GDM across quintiles of a pre-pregnancy provegetarian food pattern, using the lowest quintile as the reference category. *Results:* We identified 178 incidence cases of GDM. Women in the highest quintile (Q5) of provegetarian food pattern before pregnancy exhibited a 42% relative reduction in the odds of GDM [adjusted OR (95% CI) Q5 vs. Q1: 0.58 (0.35, 0.97); p-trend = 0.109]. Higher consumption of meat and dairy before pregnancy was associated with a significantly increased risk of GDM [adjusted OR (95% CI) Q5 vs. Q1: 1.94 (1.19, 3.16); p-trend = 0.005] and [adjusted OR (95% CI) Q5 vs. Q1: 1.77 (1.07, 2.94); p-trend = 0.082], respectively. *Conclusions:* Higher pre-pregnancy consumption of a provegetarian food pattern was associated with a lower risk of developing GDM in Spanish women. Further studies are needed to confirm these findings.

## 1. Introduction

Gestational diabetes mellitus (GDM) is diabetes first diagnosed during pregnancy [1]. GDM is the most common cause of maternal morbidity, as well as perinatal and early neonatal morbidity and mortality. This clinical condition is associated with a broad range of perinatal and pregnancy complications, including increased risk of macrosomia, cesarean delivery, neonatal hyperglycemia, hyperbilirubinemia, respiratory distress, shoulder dystocia, and hypertensive disorders of pregnancy [1,2,3]. Additionally, offspring born to women with GDM showed an increased risk of developing several chronic diseases, such as type 2 diabetes (T2D), cardiovascular disease (CVD), obesity, and negative effects on neurocognitive function [1].

The prevalence of GDM varies significantly across different populations and is influenced by several factors, such as ethnicity, maternal age, body mass index (BMI), and diagnostic criteria [4]. By 2021, the International Diabetes Federation revealed that the pooled global standardized incidence of GDM was estimated to be 14%, with 7.8% in Europe, 20.8% in South-East Asia, and the highest incidence in the Middle East and North Africa (27.6%) [4]. The increased prevalence of GDM is accompanied by a parallel increase in the prevalence of obesity, unhealthy dietary patterns, and sedentary lifestyles [1]. In this context, modifiable risk factors, such as diet—not only during pregnancy but also before pregnancy—are crucial for preventing and treating GDM [5].

In recent years, the consumption of healthy dietary patterns, such as a plant-based diet, has garnered special attention because of their potential benefits in the T2D [6], including improved insulin sensitivity, reduced inflammation, and promoted weight loss/maintenance [7,8]. However, their effects on GDM are still unclear [7]. Plant-based dietary patterns emphasize high consumption of grains, fruits, vegetables, legumes, nuts, and seeds, which are rich in fiber and antioxidants, while limiting or avoiding intake of meat and other animal products. Vegetarian diets, for example, are generally categorized into sub-types based on the group of foods that are not included in the diet, such as flexitarian (lower meat consumption), pescatarian (excludes all meats except fish and seafood), ovolactovegetarian (includes dairy and eggs), lactovegetarian (includes dairy foods), and vegan diet (excludes all animal-derived foods) [9]. Human studies evaluating the association between pre-pregnancy dietary intake and the risk of GDM are scarce [10] and mainly focused on single dietary components or nutrients, including red and processed meat intake [11], total dietary cholesterol [12], glycemic index, and fiber [13]. Since individuals consume foods as part of meals rather than as isolated foods and nutrients, evaluating dietary patterns provides an integrative vision of dietary components and their interactive effects (synergism and/or antagonism) involved in the development of diseases [14]. The analysis of priori-derived dietary scores provides an important approach to monitoring the quality of the overall diet. This approach is relevant because it is based on dietary guidelines (nutritional recommendations specific to a country or culture) and current knowledge about the healthy and unhealthy effects of the diet on the etiology of diseases [14].

The provegetarian food pattern is an a priori dietary index that prioritizes the consumption of plant-based foods instead of foods of animal origin [15]. Some studies suggested that better adherence to the provegetarian food pattern was inversely associated with mortality [15], overweight/obesity [16], and cardiometabolic risk [17]. We aimed to evaluate the association of the pre-pregnancy provegetarian food pattern with incident GDM in a Mediterranean prospective cohort of Spanish women. We hypothesized that greater adherence to the provegetarian food pattern before pregnancy could be associated with a lower risk of incident GDM.

## 2. Materials and Methods

### 2.1. Study Design and Population

This study used data from the Seguimiento Universidad de Navarra (SUN) project (www.proyectosun.es, accessed on 11 November 2024). The SUN study is an ongoing, prospective, population-based, and dynamic cohort of Spanish universities initiated in 1999. Participants are followed up every two years, using questionnaires (by post or electronic mail) to evaluate lifestyle and health-related behaviors, anthropometric measures, incident diseases, and medical/clinical conditions. Details of its design have been published elsewhere [18]. All potential candidates were informed of the right to refuse to participate or to withdraw consent at any time without reprisal. To protect the confidentiality and privacy of participants, the data were pseudonymized (each participant received a study code number). This study was conducted according to the ethical principles established by the Declaration of Helsinki. The voluntary completion of the baseline questionnaire was considered to imply informed consent. The Research Ethics Committee of the University of Navarra approved this method to request the informed consent of participants. The SUN study was approved by the Human Research Ethical Committee at the University of Navarra (091/2008 approval date 30 August 2001).

**Figure 1:**
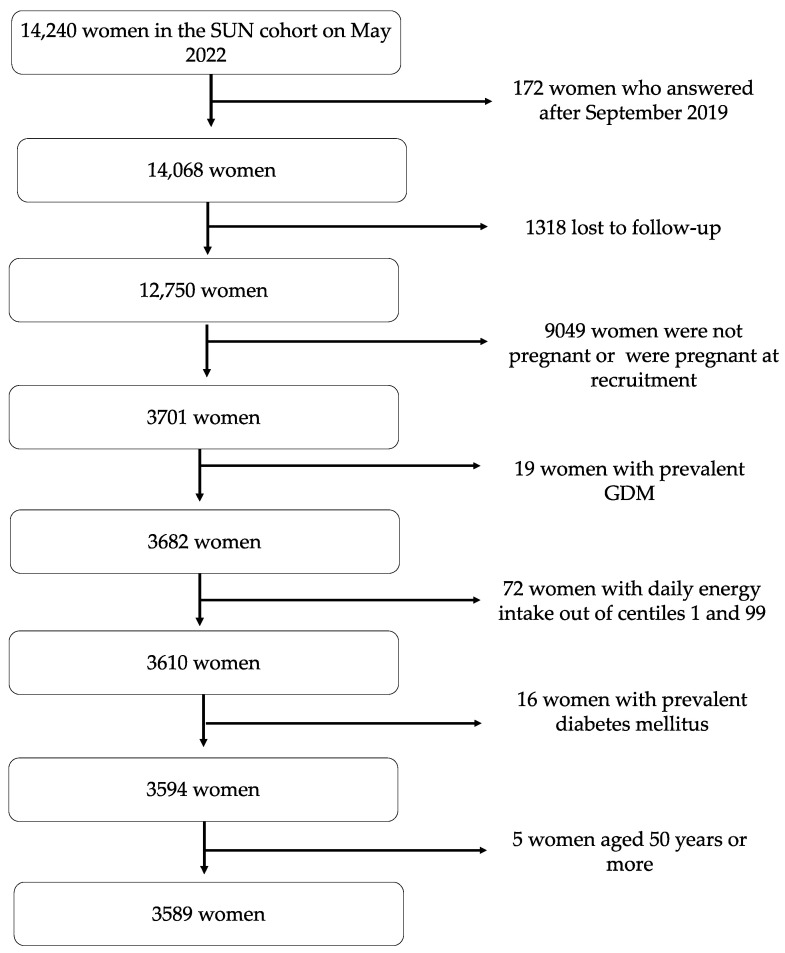
Flow chart of the study population recruited in the Seguimiento Universidad de Navarra (SUN) Project. Abbreviations: GDM, gestational diabetes mellitus.

Up to May 2022, the SUN dataset included a total of 14,240 women who had answered the baseline questionnaire. To guarantee a minimum follow-up duration of two years and nine months (enough time to complete the baseline questionnaire to reduce the risk of selection bias), we only included women recruited before September 2019 (n = 14,068). We excluded 1318 women who were lost to follow-up, 9049 women who did not become pregnant during follow-up or were pregnant at the baseline survey, 19 prevalent cases of GDM at baseline, 72 women who under-reported or over-reported values for total energy intake (limits: <1st percentile or >99th percentile), 16 prevalent cases of T2D at baseline, and 5 women aged 50 years or more (Figure 1).

### 2.2. Dietary Assessment

Dietary intake was evaluated using a semi-quantitative food frequency questionnaire (FFQ) comprising 136 food items, which had been previously validated in Spain [19,20]. Participants were required to indicate how often they ate a standard Spanish serving (servings/day) of different foods and food groups over the past year. Their consumption frequency was measured on a scale of nine categories, from “never or almost never” to “more than six servings per day”. To estimate daily consumption, the portion size was multiplied by the consumption frequency of each food item. The nutrient consumption was calculated by multiplying the consumption frequency (in grams per day) of each item by its nutrient composition, using the Spanish food composition tables [21,22].

For the main analysis, we calculated the provegetarian food pattern that has been previously described in [15]. Briefly, the provegetarian food score adjusted the consumption (g/day) of seven plant-based food groups (fruits, vegetables, potatoes, nuts, legumes, cereal grains, and olive oil) and five animal food groups (meat and meat products, eggs, fish and seafood, dairy, and animal fats) for total energy intake by using the residual method. The energy-adjusted estimates (residuals) were categorized into their quintiles. For animal food items, these quintile values were reversed: the lowest quintile was assigned a value of 5, the second quintile received a value of 4, and so on, with the highest quintile assigned a value of 1. The provegetarian food pattern consists of the sum of the quintile values of plant-based foods with the reversed quintile values of animal food items. The food items considered in the present study are shown in Table S1. The final scores assigned to participants could range from 12 for the lowest to 60 for the highest adherence (Table 1) [15].

Some studies suggest that not all plant-based foods are equally healthy [23,24]. Therefore, we distinguished between healthy and less healthy plant-based foods based on Satija’s method [23] with some modifications [16,17]. The food items included in the healthful provegetarian food pattern (hPVG) and unhealthful provegetarian food pattern (uPVG) are shown in Supplementary Table S1. We built 19 food items within categories of eight healthy plant-based food groups (vegetables, nuts, fruits, legumes, whole grains, olive oil, boiled or baked potatoes, and coffee), five less healthy plant-based food groups (refined grains, potato chips or French fries, sugar-sweetened beverages, fruit juice, and pastries), and six animal food groups (meat and meat products, eggs, fish and seafood, dairy, animal fats, and miscellaneous food). For the hPVG, the healthy plant-based food groups were scored positively. Meanwhile, the animal and less healthy plant-based food groups were scored reversely. For the uPVG, positive scores were assigned to the less healthy plant-based food groups and reversed scores to the healthy plant-based and animal food groups. To obtain the hPVG and uPVG scores, the 19 items were summed. Thus, the scores ranged from 19 for the lowest to 95 for the highest adherence (Table S1).

### 2.3. Outcome Assessment

Pregnant women who self-reported a GDM diagnosis in any of the follow-up questionnaires were requested to submit further information concerning previous glycemic disorders, the results of the oral glucose tolerance test (OGTT), insulin use during pregnancy, and medical treatment. An endocrinologist, who was unaware of dietary exposure, reviewed and determined each case of newly diagnosed GDM based on these results. There is no universal criterion for the screening and diagnosis of GDM [25]. In Spain, a two-step strategy is commonly used. The two-step strategy consisted of a 1-h initial oral test with 50 g of glucose (nonfasting) between weeks 24–28 of gestation. Those patients who tested positive performed a 3-h oral test (100 g glucose). The most common diagnostic criteria for GDM in Spain are the National Diabetes Data Group [26] and the Carpenter–Coustan criteria [27,28]. The Carpenter–Coustan criteria are: Fasting plasma glucose: 95 mg/dL (5.3 mmol/L); 1 h: 180 mg/dL (10.0 mmol/L); 2 h: 155 mg/dL (8.6 mmol/L); 3 h: 140 mg/dL (7.8 mmol/L). The National Diabetes Data Group criteria are: Fasting plasma glucose: 105 mg/dL (5.8 mmol/L); 1 h: 190 mg/dL (10.6 mmol/L); 2 h: 165 mg/dL (9.2 mmol/L); 3 h: 145 mg/dL (8.0 mmol/L). A positive test is defined as having ≥2 glucose values at or above these thresholds. For the present analyses, we only included cases that endocrinologists confirmed.

### 2.4. Assessment of Covariates

At baseline, participants completed self-administered questionnaires to obtain information about sociodemographic characteristics (e.g., sex, marital status, years of university education, etc.), lifestyle (e.g., physical activity, smoking habits, sedentary behaviors, snacking, ongoing special diet, etc.), anthropometric measurements (weight and height), and family and personal medical history. Non-communicable diseases (NCDs) such as cancer, diabetes, and cardiovascular events were confirmed by medical reports. Self-reported anthropometric measurements and diagnosis were previously validated in a subsample, reporting high validity [29,30]. To evaluate physical activity, we used the validated Spanish version of the Harvard Nurses’ Health Study physical activity questionnaire, which included 17 items [31]. Leisure time activities were quantified in metabolic equivalent tasks (METs) per week by assigning the typical energy expenditure to each activity and multiplying it by the time spent in hours per week for each activity [31]. Adherence to the Mediterranean diet (9-point score) was performed following the method outlined by Trichopoulou et al. [32,33].

### 2.5. Statistical Analysis

Baseline characteristics of participants are presented overall and across quintiles of provegetarian food patterns. Data are presented as means ± standard deviations (SD) for quantitative variables and percentages and numbers for categorical variables. The association between the provegetarian food patterns (including the hPVG and uPVG) and the risk of GDM was assessed by logistic regression, obtaining odds ratios (OR) and their 95% confidence intervals (CI), with the lowest quintile as the reference category. We adjusted for selected variables described in the literature instead of adhering to statistical criteria to identify potential confounding factors [34,35]. We evaluated two multivariable-adjusted models. Model 1 was adjusted for maternal age at first pregnancy or the diagnosis of GDM at the cohort (years, continuous) and pre-pregnancy BMI (kg/m^2^, continuous). Model 2 was further adjusted for the time between recruitment and the first pregnancy (continuous), university education (years; 3–4, 5–6, >6), smoking habits (never, former, and current), physical activity (metabolic equivalent h/wk; tertiles), family history of diabetes (yes, no), number of pregnancies during follow-up (1, 2, ≥3), parity (nulliparous, 1, 2, ≥3, missing), time spent watching TV (hours/d; tertiles), hypertension (yes, no), following a special diet at baseline (yes, no), snacking (yes, no), and total energy intake (kcal/d; tertiles). To evaluate the linear trend, we assigned the median value of each quintile of the provegetarian food patterns and used it as a continuous variable. The robustness of our findings was evaluated by sensitivity analyses in different scenarios: (a) considering different plausible energy limits proposed by Willett [36]; (b) excluding women < 30 years at maternal age; (c) excluding participants diagnosed with CVD or cancer; (d) excluding participants following a special diet; (e) excluding women with previous pregnancies at baseline; (f) excluding women whose first pregnancy occurred 10 years after recruitment; and (g) excluding women with pregnancies occurring early during the follow-up period (first two years). Each provegetarian food group (grams per day) was energy-adjusted using the residual method and then converted into quintiles to evaluate its association with GDM incidence. All statistical analyses were conducted using the software Stata 18 (StataCorp), and *p*-values < 0.05 were considered significant.

## 3. Results

### 3.1. Study Sample Characteristics

Baseline characteristics of women according to pre-pregnancy quintiles of the provegetarian food pattern are shown in Table 2. During a median of 14.6 years follow-up (1999–2022), 178 cases of GDM were reported among 3589 pregnant women with a mean age of 28 (SD: 4.3) years (mean (SD) age at baseline). Women with a greater pre-pregnancy provegetarian food pattern (Q5) were older, less likely to be smokers, had a higher prevalence of family history of diabetes, engaged in more physical activity, were less likely to eat between-meals, and had better adherence to the Mediterranean diet compared to women in the first quintile (Q1) (Table 2). Similar results were found in women with the highest adherence (Q5) to the hPVG compared with those in the lowest adherence group (Q1) (Table S2). In contrast, women in the highest quintile of the uPVG were more likely to be younger, had a lower prevalence of family history of diabetes, were less physically active, were more likely to eat between meals, and presented lower adherence to the Mediterranean diet than women in the lowest quintile (Table S2).

Women in the highest quintile (Q5) of the provegetarian food pattern had lower consumption of fats (% of energy), sodium, meat and fish, animal fat, dairy products, and eggs but had a higher intake of cereal grains, vegetables, fruits, nuts, and olive oil across quintiles (Table 3). Similarly, those women with the highest hPVG score had higher consumption of vegetables, boiled potatoes, fruits, legumes, nuts, whole grains, olive oil, coffee and lower intake of fats (% of energy), sodium, potato chips/ French fries, dairy, eggs, meat, pastries, and sugar-sweetened beverages compared with women in the lowest hPVG quintile (Table S3). Additionally, they had higher energy intake and higher carbohydrate (% of energy) consumption (Table S3). On the contrary, women in the fifth quintile of the uPVG showed higher energy intake and saturated fatty acid (SFA) (% of energy) (Table S4). Furthermore, they also had a higher consumption of sodium and less healthy plant-based foods (French fries, fruit juice, refined grains, sugar-sweetened beverages, and pastries) compared to those in the lowest adherence group (Q1) (Table S4).

### 3.2. Association Between the Provegetarian Food Patterns and Their Components Before Pregnancy and the Risk of GDM

The ORs for GDM according to quintiles of the provegetarian food pattern before pregnancy are presented in Table 4. No statistically significant association was found between the highest provegetarian food pattern category (Q5) vs. the lowest (Q1: reference) and the risk of GDM in the unadjusted model [(OR: 0.65; 95% CI: 0.40–1.07; p-trend = 0.211)] and the multivariable logistic regression (model 1) adjusted for age (at first pregnancy at the cohort or GDM diagnosis) and pre-pregnancy BMI [(OR: 0.67; 95% CI: 0.40–1.10; p-trend = 0.252)]. However, in the fully adjusted multivariable logistic regression (model 2), higher adherence to the pre-pregnancy provegetarian food pattern (Q5) was significantly associated with decreased odds of GDM [(OR: 0.58; 95% CI: 0.35–0.97)] compared to the lowest quintile (Q1). Nonetheless, no significant inverse linear trend across increasing quintiles was found (p-trend = 0.109). The association between hPVG, uPVG and GDM is shown in Tables S6 and S7, respectively. The fully multivariable-adjusted models revealed no significant association between the highest intake of hPVG [(OR _Q5 vs. Q1_: 0.63; 95% CI: 0.35–1.12; p-trend = 0.075)] and uPVG [(OR _Q5 vs. Q1_: 1.13; 95% CI: 0.67–1.91; p-trend = 0.789)] before pregnancy and the prevalence of GDM.

**Figure 2:**
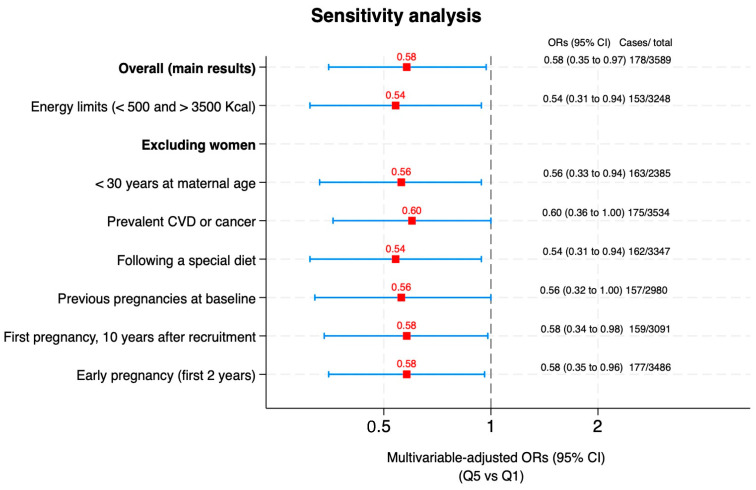
Sensitivity analyses for the association between the provegetarian food pattern and the risk of gestational diabetes before pregnancy (multivariable-adjusted ORs and 95% CI for the highest vs. lowest quintile). Abbreviations: Ref, reference; Q, quintiles. Values are odds ratios (OR) and 95% confidence intervals.

The results of the sensitivity analyses, in which some of our assumptions were modified, or new potential confounders were introduced (described previously), did not substantially change (Figure 2).

The relationship between the consumption of each component of the provegetarian food pattern and the development of GDM is shown in Table 5. We found that women in the highest quintile of dairy product consumption before pregnancy showed an increased risk of developing GDM compared to those in the lowest quintile [OR (95% CI) _Q5 vs. Q1_: 1.77 (1.07–2.94); p-trend = 0.082], but no linear trend among quintiles was found. Furthermore, greater pre-pregnancy intake of meat and meat products was associated with increased odds of GDM in comparison with the lowest quintile [OR (95% CI) _Q5 vs. Q1_: 1.94 (1.19–3.16)], showing a linear dose-response relationship across quintiles (p-trend = 0.005). No significant positive associations were observed between GDM and the other provegetarian food groups (Table 5).

## 4. Discussion

In this study, greater adherence to the provegetarian food pattern before pregnancy was associated with a lower risk of developing GDM in a Mediterranean cohort. Furthermore, a higher intake of pre-pregnancy dairy, as well as meat and meat products, was positively associated with the risk of GDM. Our results were consistent with previous studies evaluating the adherence to plant-based diets and the risk of GDM in prospective [37,38,39] and case-control studies [40,41]. In the Nurses’ Health Study II (1991–2001), higher pre-pregnancy adherence to the overall plant-based diet index (PDI) and the healthful plant-based diet was inversely associated with a lower risk of GDM [37]. However, the unhealthful plant-based diet was not significantly associated with GDM. In our study, the highest adherence to the hPVG before pregnancy was no longer significant with GDM, although the magnitude of the effect was quite similar to that observed in the provegetarian food pattern. We also found a nonsignificant direct association between uPVG and GDM incidence. In the same line, in the Tongji Maternal and Child Health cohort study, two studies evaluated adherence to overall PDI [38] and the Chinese Dietary Guidelines Compliance Index for Pregnant Women (CDGCI-PW) [39] regarding their impact on GDM and maternal complications. In this cohort, women were recruited during their first prenatal evaluation (within 8–16 weeks of gestation). Wang et al. showed that higher adherence to the overall PDI (Q4 vs. Q1) was positively associated with a 57% reduced odds of GDM. When investigators explored the PDI quality (healthy and unhealthy), only the healthy PDI was significantly associated with a reduced risk of developing GDM [38]. Moreover, Ding et al. observed that greater compliance with the CDGCI-PW was linked to a 70% lower risk of gestational hypertension and a 62% reduction in GDM risk [39]. Olmedo-Requena et al. observed similar findings in a case-control study performed among Spanish pregnant women (291 GDM cases and 1175 controls) [40]. Women with higher pre-pregnancy adherence to the Mediterranean diet had a strongly reduced risk of GDM [40]. Similarly, a case-control study revealed that higher consumption of an unhealthy dietary pattern (mainly ultra-processed foods) was directly associated with the risk of GDM in Iranian pregnant women [41]. A similar trend was observed in a recent meta-analysis that included 32,006 participants from 10 studies conducted in several countries. Results showed that plant-based diets are associated with a lower risk of GDM (fixed effects pooled effect size [RR = 0.88; 95% CI: 0.81–0.96, I^2^ = 14.8%]). This association was slightly stronger in healthy plant-based diets compared to unhealthy diets [7]. Staple foods (minimally processed), such as whole grains, vegetables, fruits, legumes, and nuts, confer protective effects on health outcomes. In fact, the Global Burden of Disease 2021 report indicated that low intake of fruits, grains, vegetables, and fiber is associated with an increased risk of mortality [42]. The provegetarian food pattern does not discriminate between healthy and unhealthy plant-based foods. For instance, the potato (mashed, boiled, and fried) item scored positively in the provegetarian food pattern. Some studies suggested the relationship between potato consumption, in particular French fries, and the risk of GDM [43,44]. However, in our cohort, uPVG did not reach statistical significance.

Plant-based foods vary widely in nutritional quality and have different impacts on health [23,45]. Discrepancies in findings regarding the consumption of unhealthy diet patterns and the risk of developing diabetes mellitus could be attributable to the group of foods included, which varies in nutritional composition, density, type, and degree of industrial processing [46,47]. However, it is important to point out that a greater intake of ultra-processed foods is closely related to an increased risk of T2D [47] and GDM [48,49]. In this context, the consumption of a healthy plant-based diet could have a positive impact on preventing GDM.

Another study evaluated a posteriori dietary patterns (determined by factor analysis) and their impact on GDM in an Australian population-based prospective cohort study [50]. A Mediterranean-style pattern before pregnancy was inversely associated with a 15% lower risk of developing GDM for each SD increase in the score [50]. Similar results were found in the St. Carlos GDM prevention clinical trial, which reported a 25% lower incidence of GDM in pregnant women assigned to a Mediterranean diet supplemented with extra-virgin olive oil and pistachios compared with those assigned to the control diet (standard Mediterranean diet with limited fat intake) [51]. Moreover, pregnant women allocated to the intervention group showed better improvements in maternal and neonatal outcomes [51]. There are differences between the provegetarian food pattern and the traditional Mediterranean diet. Despite both dietary patterns being rich in plant-based foods, the Mediterranean diet also includes moderate consumption of dairy products, moderate-to-high consumption of fish, and low consumption of meat and meat products [33]. Meanwhile, fish consumption is a negative score in the provegetarian food pattern. In our study, the provegetarian food pattern was moderately correlated (rho = 0.49) with the traditional Mediterranean diet proposed by Trichopoulou et al. [32,33]. These findings suggest that adopting a provegetarian food pattern could be an optimal choice for transitioning to sustainable food systems and encouraging its adoption.

GDM is a complex and multifactorial condition, which increases the risk of short- and long-term adverse outcomes in both mothers and their offspring [1,2]. Several biological mechanisms have been proposed to explain the effect of plant-based diets on GDM [52]. The provegetarian food pattern prioritizes the consumption of vegetables-derived foods rich in (poly) phenols, fiber, micronutrients, and healthy fatty acids, improving glucose and lipid metabolism with anti-inflammatory and antioxidant properties that could reduce oxidative stress [6,52]. Plant-based diets contain several bioactive compounds, which may exert their biological activity by regulating gene expression related to lipogenesis and glucose metabolism, gut microbiota, and endothelial function, as well as downregulating the synthesis of pro-inflammatory cytokines [52,53]. The overproduction of reactive oxygen species due to increased levels of reactive nitrogen species triggers the risk of fetal malformations [54]. In GDM, oxidative stress induces nitric oxide overload, causing platelet dysfunction and membrane damage and promoting increased lipid peroxidation [54]. In this context, plant-based diets could be considered the cornerstone of GDM prevention and management [8].

Some studies suggested that the protective effects of higher adherence to plant-based dietary patterns and the lower risk of T2D and GDM might be, at least in part, explained by their positive impact on weight reduction/control, in which BMI could serve as a confounder and mediator [7,8]. This point is relevant, considering that being overweight or obese before pregnancy is one of the most significant GDM risk factors [1]. In our study, there was no alteration of the effect estimates after controlling for pre-pregnancy BMI. These results can be explained by a lower prevalence of overweight and obesity (8%) in our sample, and this is a unique feature of our cohort that highlights the novelty of our findings, not apparently mediated by changes in BMI. Despite this, it is recognized that greater adherence to plant-based diets could have a synergistic impact (direct or indirect effect) on reducing overweight and the risk of developing diabetes mellitus [7,55].

Few studies have evaluated the relationship between vegetarian diets and GDM, but findings are still unclear [56,57]. A study recruited 714 pregnant women (at 5–16 weeks of gestation) in South India, identifying 157 cases of GDM [56]. By the combination of a priori and a posteriori methods, three pre-pregnancy dietary patterns were identified, including the high-diversity urban (HDU) pattern (higher diversity group of foods, including vegetarian and non-vegetarian, home-cooked traditional dishes), the rice-fried snacks–chicken–sweets (RFCS) pattern (higher intake of rice dishes, sugar, snacks, and sweets), and the healthy traditional vegetarian (HTV) pattern (mainly includes plant-based foods and fermented dairy products). A significant inverse association between the HDU dietary pattern and the risk of GDM was found, but this association weakened after BMI adjustment. No significant association between the HTV and RFCS diets and the risk of GDM was observed [56]. Another study examined the association between following a vegetarian diet during pregnancy and neonatal outcomes using data from the Eunice Kennedy Shriver National Institute of Child Health and Human Development’s Fetal Growth Studies–Singletons (included women from four ethnicities) [57]. Findings indicated that full-vegetarian diets (vegans and ovolactovegetarians) during pregnancy were associated with an increased risk of delivering a small-for-gestational-age neonate but not related to postnatal morbidity [57]. In contrast, maternal intake of a plant-based diet was associated with lower birth weight in women of Caucasian ethnicity and higher birth weight in women of Asian ethnicity in a prospective study covering women from different ethnic groups living in Canada (3997 full-term mother–infant pairs) [58]. Discrepancies in these findings may be partly explained by the complex nature of dietary patterns, the greater variability in the definition of a posteriori plant-based diets, and their varied effects of plant-based diets across ethnic groups [7,58].

When we analyzed each component of the provegetarian food pattern (with no mutual adjustment for each other), higher consumption of dairy and meat was associated with a 1.8-fold and 1.9-fold increase in the risk of developing GDM, respectively. In terms of foods, the association between higher intake of dairy products and diabetes incidence remains controversial [59,60,61]. For example, no significant relationship between dairy consumption and GDM was found in the National Health and Nutrition Examination Survey (NHANES, 2013–2016) [59]. However, the assessment of dairy consumption was based on a 1-day dietary record, which is unable for the regular intake estimation. In contrast, in the Nutrition in Pregnancy and Growth in Southwest China (NPGSC) prospective cohort study, investigators revealed that a higher intake of dairy products in mid-pregnancy was associated with a higher risk of GDM [60]. Alvarez-Bueno et al. evaluated dose–response dairy consumption using forest plots and summarized the relative risk ratios (RRs) reported by meta-analyses (low vs. high consumption) and the risk of T2D [61]. The majority of the studies reported that dairy and low-fat dairy products were inversely associated with T2D. Four out of five studies revealed that an intake of 200–400 g/d of total dairy product consumption (RR range: 0.93–0.97) was associated with a lower GDM risk [61]. In our sample, it is noteworthy that women categorized into the highest quintile of dairy products had a greater mean consumption of dairy (857 g/day) compared to previous studies in which dairy products were reported to reduce the risk of T2D [61]. Nevertheless, this evidence should be interpreted with caution because studies used different diagnostic criteria for GDM, potentially misclassified dairy intake, and included other factors that could contribute to making difficult direct comparisons.

There has been evidence to support that meat intake increases the risk of GDM [62,63]. A recent meta-analysis of individual participant data from 31 cohorts in 20 countries indicated that higher consumption of meat was associated with an increased risk of developing T2D. Furthermore, 50 g/day of processed meat increased (HR: 1.15; 95% CI: 1.11–1.20; I^2^ = 59%) the risk of T2D by 15% [62]. A previous study in the SUN cohort demonstrated that higher meat consumption before pregnancy was significantly associated with a higher risk of GDM in a dose–response manner [OR (95% CI) _Q4 vs. Q1_: 1.67 (1.06–2.63); p-trend = 0.008] [63]. These findings are consistent with other studies [10,60]. Several possible mechanisms could be involved in the adverse effects of higher meat intake on GDM, mainly influenced by meat components. Red and processed meats are high in saturated fats, iron, and advanced glycation end products (cooked at high temperatures), which disrupt insulin and inflammatory pathways, contributing to insulin resistance and oxidative stress [62,64].

Despite the increased trend in the consumption of plant-based diets, self-identification tends to overestimate the number of vegetarians compared to definitions based on their dietary intake [57,65]. Recently, a study examined the prevalence of plant-based diets obtained from weekly food intake frequency in the Spanish population, using data from the Spanish National Health Survey (2001–2017) [65]. That study revealed that the adoption of plant-based diets increased across the years, but their prevalence still remains low [65].

Our research revealed a significant 42% reduction in the risk of GDM among Spanish women who adhered to the provegetarian food pattern before pregnancy. From a public health point of view, government policies urge the reinforcement of the transition to plant-forward food patterns at the population level. The provegetarian food pattern, centered around consuming mainly plant foods, offers a pragmatic approach for the general population and serves as a stepping stone toward vegetarianism. A shift toward the provegetarian food pattern, encouraging the consumption of minimally processed plant-based foods, may improve health outcomes and promote a sustainable approach to address the ecological impact of animal-based foods [66]. This study presents valuable findings that can guide the implementation of GDM counseling in primary care.

The strengths of our study include its prospective design with a large sample size of individuals from a Mediterranean country. The low average BMI of our participants and the lack of attenuation of the effect of the dietary pattern after controlling for BMI represent a novel finding. The SUN cohort collects detailed and comprehensive potential confounders and uses well-validated measurements, which were included in our analysis. However, there are some limitations to consider.

First, we have no information on diet during pregnancy. Nonetheless, pre-pregnancy diet is a critical factor for maternal and infant health and the development of GDM [1,5]. Future research should be conducted to evaluate the effects of the provegetarian food pattern during pregnancy on the risk of GDM.

Second, our study is based on a cohort of Spanish university graduates, which may limit the generalizability of our findings to women of other ethnicities and socioeconomic backgrounds. However, this restriction (university graduates) enhances the internal validity of our results by ensuring a homogeneous educational and socioeconomic level, thereby reducing potential confounding from these variables. Evidence suggested that low-income population groups tend to consume fewer fruits, vegetables, and whole grains due to the higher economic cost involved [67], and therefore, replication of our findings in different socioeconomic groups would be valuable.

Third, the use of the FFQ as a method to evaluate dietary habits may be prone to some measurement errors. Nonetheless, the FFQ used in this study was validated in the Spanish population and replicated in other cohorts with consistent results [19,20]. Furthermore, the FFQ contemplates the foods that are frequently consumed in Spain.

Fourth, the classification of GDM in the SUN cohort differs from other studies, which could impact the results. Finally, due to the observational nature of the SUN cohort, residual or unmeasured confounding cannot be completely ruled out.

## 5. Conclusions

Greater pre-pregnancy adherence to the provegetarian food pattern was associated with a reduced risk of GDM in Spanish women. Our findings have significant potential to enhance the quality of preconception nutrition counseling and offer an alternative dietary approach to reduce the prevalence of GDM in Spanish women. However, given the disparities in findings from previous studies considering different ethnic groups, further research is needed to investigate the relationship between a provegetarian diet before and during pregnancy in different populations.

## Figures and Tables

**Table 1:** Scoring criteria for the provegetarian food pattern.

Provegetarian Food Pattern (Range of 12–60)	
Component	Criteria
Plant-based food groups	Energy-adjusted quintiles
Vegetables	Positive
2. Fruits	Positive
3. Legumes	Positive
4. Cereal grains	Positive
5. Potatoes	Positive
6. Nuts	Positive
7. Olive oil	Positive
Animal food groups	
8. Dairy	Reverse
9. Eggs	Reverse
10. Meat and meat products	Reverse
11. Fish and seafood	Reverse
12. Animal fat	Reverse

**Table 2:** Baseline characteristics of pre-pregnancy study population according to quintiles of the provegetarian food pattern.

	Quintiles of the Provegetarian Food Pattern Before Pregnancy				
	Q1 19–32	Q2 33–35	Q3 36–37	Q4 38–40	Q5 41–53
Participants, n	891	765	580	698	655
Mean of the provegetarian food pattern	29.5 (2.5)	34.1 (0.8)	36.5 (0.5)	38.9 (0.8)	43.5 (2.5)
Age at baseline (years)	27.4 (4.3)	27.6 (4.3)	27.8 (4.3)	27.7 (4.2)	28.0 (4.2)
BMI at baseline (kg/m^2^)	21.3 (2.7)	21.4 (2.7)	21.4 (2.7)	21.3 (2.4)	21.2 (2.5)
Physical activity (METs/h/wk)	18.3 (21.6)	18.4 (18.8)	19.1 (19.9)	19.1 (19.5)	21.9 (21.1)
Parity (%)					
Nulliparous	81.4	85.0	82.2	84.0	82.7
1 pregnancy	9.4	6.9	6.4	9.0	9.5
2 pregnancies	3.9	3.9	4.8	2.9	2.9
≥3 pregnancies	2.5	3.1	3.1	1.4	1.5
Missing	2.8	1.0	3.4	2.7	3.4
Family history of diabetes mellitus (%)	9.3	11.1	8.4	10.5	11.6
Hypertension (%)	1.1	1.3	0.5	1.3	1.2
Smoking status (%)					
Never	53.8	56.2	57.8	59.0	59.2
Current	28.2	24.2	26.4	23.4	24.0
Former	18.1	19.6	15.9	17.6	16.8
Alcohol intake (g/day)	3.7 (5.1)	4.0 (6.3)	3.6 (4.7)	4.0 (5.1)	3.8 (4.4)
Pregnancies during Follow-up (%)					
1 pregnancy	46.9	47.3	46.2	48.7	50.7
2 pregnancies	32.9	34.0	33.1	31.4	30.8
≥3 pregnancies	20.2	18.7	20.7	19.9	18.5
Between-meal snacking (%)	47.1	42.2	40.5	39.7	41.2
Following special diets (%)	5.8	5.8	5.7	7.7	9.0
Television viewing (h/day)	1.6 (1.2)	1.7 (1.3)	1.6 (1.2)	1.7 (1.3)	1.6 (1.3)
Years at university	4.7 (1.2)	4.7 (1.2)	4.7 (1.2)	4.7 (1.2)	4.7 (1.2)
Adherence to MedDiet (0–9 points)	2.9 (1.5)	3.6 (1.5)	3.9 (1.6)	4.6 (1.5)	5.2 (1.5)

Abbreviations: METs, metabolic equivalents; MedDiet, Mediterranean diet.

**Table 3:** Dietary intake of pre-pregnancy study population according to quintiles of the provegetarian food pattern.

	Quintiles of the Provegetarian Food Pattern before Pregnancy				
	Q1 19–32	Q2 33–35	Q3 36–37	Q4 38–40	Q5 41–53
Participants, n	891	765	580	698	655
Mean of the provegetarian food pattern	29.5 (2.5)	34.1 (0.8)	36.5 (0.5)	38.9 (0.8)	43.5 (2.5)
Total energy intake (kcal/day)	2573 (764)	2452 (749)	2438 (713)	2405 (679)	2541 (756)
Carbohydrate (% of energy)	40 (7)	43 (7)	44 (7)	45 (7)	47 (6)
Protein (% of energy)	19 (3)	19 (3)	18 (3)	18 (3)	17 (3)
Fat (% of energy)	40 (6)	38 (6)	37 (7)	36 (7)	35 (6)
SFA (% of energy)	15 (3)	13 (3)	13 (3)	12 (3)	11 (3)
TFA (% of energy)	0.5 (0.2)	0.4 (0.2)	0.4 (0.2)	0.3 (0.2)	0.3 (0.1)
PUFA (% of energy)	5.3 (1.6)	5.4 (1.9)	5.4 (1.7)	5.3 (1.6)	5.4 (1.7)
MUFA (% of energy)	17 (4)	16 (4)	16 (4)	16 (4)	16 (4)
Total dietary fibre intake (g/day)	19 (9)	21 (9)	23 (10)	25 (10)	30 (12)
Sodium intake (mg/day)	3954 (2356)	3641 (4096)	3470 (2117)	3290 (1952)	3332 (2023)
Food group intake (g/day)					
Vegetables	472 (327)	512 (296)	556 (347)	605 (329)	724 (411)
Potatoes	48 (45)	54 (57)	54 (48)	56 (46)	67 (53)
Fruits	237 (253)	293 (269)	334 (339)	381(306)	438 (329)
Legumes	19 (17)	20 (12)	22 (19)	24 (17)	28 (20)
Nuts	4 (6)	5 (8)	6 (10)	7 (13)	12 (17)
Cereals grains	81 (56)	94 (75)	106 (69)	109 (68)	131 (77)
Olive oil	16 (14)	18 (14)	20 (15)	22 (15)	26 (17)
Animal fat	1.7 (2.9)	1.0 (2.3)	1.0 (2.6)	0.7 (2.1)	0.5 (1.8)
Dairy	611 (329)	497 (261)	446 (249)	420 (231)	358 (216)
Fish and seafood	111 (85)	100 (72)	93 (51)	97 (61)	90 (67)
Meat and meat products	227 (102)	194 (89)	181 (75)	164 (74)	146 (73)
Eggs	28 (20)	25 (14)	22 (13)	21 (15)	17 (11)

Abbreviations: SFA, saturated fatty acid; TFA, trans fatty acid; PUFA, polyunsaturated fatty acid; MUFA, monounsaturated fatty acid.

**Table 4:** ORs (95% CIs) for gestational diabetes mellitus according to quintiles of the provegetarian food pattern before pregnancy.

	Quintiles of the Provegetarian Food Pattern Before Pregnancy					
	Q1 19–32	Q2 33–35	Q3 36–37	Q4 38–40	Q5 41–53	p-Trend
Median score	30	34	36	39	43	
Nº cases/total	51/891	36/765	26/580	40/698	25/655	
Crude model	1.0 (Ref.)	0.81 (0.52, 1.26)	0.77 (0.48, 1.25)	1.00 (0.65, 1.53)	0.65 (0.40, 1.07)	0.211
Model 1	1.0 (Ref.)	0.85 (0.54, 1.33)	0.76 (0.46, 1.26)	1.03 (0.67, 1.60)	0.67 (0.40, 1.10)	0.252
Model 2	1.0 (Ref.)	0.86 (0.55, 1.34)	0.74 (0.44, 1.23)	1.02 (0.66, 1.60)	**0.58 (0.35, 0.97)**	0.109

Abbreviations: Ref, reference. Values are odds ratios (OR) and 95% confidence intervals. Crude model: Unadjusted model; Model 1: Model adjusted for maternal age at first pregnancy or GDM diagnosis at the cohort (continuous) and pre-pregnancy BMI (continuous); Model 2: Model 1 + time between recruitment and the first pregnancy (continuous), university education (years; 3–4, 5–6, >6), smoking status (never, former, and current), physical activity (metabolic equivalent h/wk; tertiles), family history of diabetes (yes, no), number of pregnancies during the follow-up (1, 2, ≥3), parity (nulliparous, 1, 2, ≥3, missing), time spent watching TV (hours; tertiles), hypertension (yes, no), following a special diet at baseline (yes, no), snacking (yes, no), total energy intake (kcal/d; tertiles).

**Table 5:** ORs (95% CIs) for gestational diabetes mellitus according to quintiles of each component of the provegetarian diet before pregnancy.

	Energy-Adjusted Quintiles of Consumption Before Pregnancy of Each Food Item Included in the Definition of the Provegetarian Diet and Risk of Gestational Diabetes Mellitus					
	Q1	Q2	Q3	Q4	Q5	p-Trend
Vegetables	1.0 (Ref.)	1.14 (0.70, 1.87)	0.79 (0.47, 1.33)	1.13 (0.70, 1.82)	1.08 (0.65, 1.78)	0.762
Fruits	1.0 (Ref.)	1.36 (0.81, 2.28)	1.57 (0.94, 2.60)	1.19 (0.70, 2.03)	1.11 (0.64, 1.93)	0.843
Legume	1.0 (Ref.)	0.84 (0.50, 1.41)	1.20 (0.73, 1.96)	1.00 (0.60, 1.68)	1.23 (0.75, 1.99)	0.302
Cereals	1.0 (Ref.)	1.40 (0.87, 2.26)	1.35 (0.83, 2.20)	0.91 (0.53, 1.55)	0.74 (0.44, 1.26)	0.063
Potatoes	1.0 (Ref.)	0.89 (0.54, 1.47)	1.03 (0.61, 1.72)	0.88 (0.54, 1.42)	0.73 (0.44, 1.19)	0.196
Olive oil	1.0 (Ref.)	1.19 (0.71, 1.98)	1.57 (0.94, 2.63)	1.04 (0.63, 1.70)	0.75 (0.43, 1.30)	0.122
Nuts	1.0 (Ref.)	1.05 (0.64, 1.74)	1.03 (0.60, 1.76)	1.29 (0.75, 2.22)	1.16 (0.71, 1.90)	0.571
Dairy	1.0 (Ref.)	1.67 (1.01, 2.77)	0.97 (0.55, 1.70)	1.33 (0.79, 2.23)	**1.77 (1.07, 2.94)**	0.082
Eggs	1.0 (Ref.)	0.99 (0.59, 1.68)	0.96 (0.58, 1.59)	1.05 (0.61, 1.80)	1.17 (0.70, 1.95)	0.662
Meat and meat products	1.0 (Ref.)	1.24 (0.74, 2.08)	1.06 (0.62, 1.83)	1.54 (0.93, 2.56)	**1.94 (1.19, 3.16)**	0.005
Fish and seafood	1.0 (Ref.)	1.61 (0.95, 2.73)	1.62 (0.95, 2.74)	1.24 (0.72, 2.12)	1.10 (0.64, 1.90)	0.631
Animal fat	1.0 (Ref.)	0.94 (0.49, 1.79)	**0.49 (0.24, 0.99)**	0.76 (0.44, 1.31)	0.68 (0.39, 1.18)	0.365

Abbreviations: Ref, reference; Q, quintiles. Values are odds ratios (OR) and 95% confidence intervals. Multivariable-adjusted model: adjusted for maternal age at first pregnancy or GDM diagnosis within the cohort (continuous), pre-pregnancy BMI (continuous), time between recruitment and the first pregnancy (continuous), university education (years; 3–4, 5–6, >6), smoking status (never, former, and current), physical activity (metabolic equivalent h/wk; tertiles), family history of diabetes (yes, no), number of pregnancies during the follow-up (1, 2, ≥3), parity (nulliparous, 1, 2, ≥3, missing), time spent watching TV (hours; tertiles), hypertension (yes, no), following a special diet at baseline (yes, no), snacking (yes, no), and total energy intake (kcal/d; tertiles).

## Data Availability

Information and data from this study are available on request from Maira Bes-Rastrollo (mbes@unav.es).

## Supplementary Materials

The following supporting information can be downloaded at: https://www.mdpi.com/article/10.3390/medicina60111881/s1, Table S1: Food groups and items included in the provegetarian food patterns and score criteria for a hPVG and uPVG pattern from the SUN cohort FFQ; Table S2: Baseline characteristics of pre-pregnancy study population according to quintiles of the healthy provegetarian food pattern, Table S3: Baseline characteristics of pre-pregnancy study population according to quintiles of the unhealthy provegetarian food pattern, Table S4: Dietary intake of pre-pregnancy study population according to quintiles of the healthy provegetarian food pattern, Table S5: Dietary intake of pre-pregnancy study population according to quintiles of the unhealthy provegetarian food pattern, Table S6: ORs (95% CIs) for gestational diabetes mellitus according to quintiles of the healthy provegetarian food pattern before pregnancy, Table S7: ORs (95% CIs) for gestational diabetes mellitus according to quintiles of the unhealthy provegetarian food pattern before pregnancy.
