# Supplementary material for: Pre-Pregnancy Provegetarian Food Pattern and the Risk of Developing Gestational Diabetes Mellitus: The Seguimiento Universidad de Navarra (SUN) Cohort Study
Source: Medicina (Kaunas). 2024 Nov 16;60(11):1881. doi: 10.3390/medicina60111881 (PMC11596851; doi:10.3390/medicina60111881)
Supplement: Supplementary file 1 [file medicina-60-01881-s001.zip › medicina-3257049-supplementary.pdf]

medicina MDPI

Supplementary Materials

Table S1. Food groups and items included in the provegetarian food patterns and score criteria for a hPVG and uPVG pattern from the SUN cohort FFQ

**Plant- based Food groups**	**Items**	**Healthy provegetarian food pattern (hPVG)**	**Unhealthy provegetarian food pattern (uPVG)**
1. Vegetables	Swiss chard, spinach, cabbage, cauliflower, broccoli, lettuce, chicory, tomatoes, carrot, pumpkin, green beans, eggplant, zucchini, cucumber, pepper, asparagus, others	Positive	Reverse
2. Fruits	Citrus, grapes, banana, apple, pear, strawberry, peach, apricot, nectarine, cherries, plums, figs, melon, watermelon, grapes, mango, papaya, kiwi, olives, avocado, canned fruit*	Positive	Reverse
3. Legumes	Lentils, chickpeas, beans, peas	Positive	Reverse
4. Nuts	Almonds, peanuts, hazelnuts, walnuts	Positive	Reverse
5. Olive oil	Olive oil	Positive	Reverse
6. Whole grains	Whole-grain bread	Positive	Reverse
7. Refined grains	Breakfast cereals, white bread, white rice, pasta,	Reverse	Positive
8. Boiled/ baked potato	Boiled or baked potatoes	Positive	Reverse
9. Potato chips /French fries	Potato chips or French fries	Reverse	Positive
10. Coffee	Coffee, decaffeinated coffee	Positive	Reverse
11. Fruit juices	Fresh orange juice, other natural fruit juices	Reverse	Positive
12. Sugar-sweetened beverages	Sugar-sweetened beverages, bottled fruit or vegetable juices	Reverse	Positive
13. Pastries	Cookies, home-baked and ready-made cakes, muffins, donuts, croissant, churros, cakes, chocolates, nougat, marzipan	Reverse	Positive
**Animal Food Groups**	**Items**		

14. Dairy	Whole milk, skim or low-fat milk, condensed milk, cream, milk shake, yogurt, custard, cheese, ice cream	Reverse	Reverse
15. Eggs	Eggs	Reverse	Reverse
16. Meat and meat products	Beef, veal, pork, lamb, liver, other viscera, chicken, turkey, serrano ham, cooked ham, chorizo, salami, mortadella, foie gras, black pudding, bacon, other cured or smoked meats, hamburger, hot dog	Reverse	Reverse
17. Fish and seafood	White fish, blue fish, salted or smoked fish, clams, mussels, shrimp, squid, octopus	Reverse	Reverse
18. Animal fat	Butter, lard	Reverse	Reverse
19. Miscellaneous food	Pizza, instant soups, mayonnaise	Reverse	Reverse

Abbreviations: FFQ, food frequency questionnaire; hPVG, healthy provegetarian food pattern; uPVG, unhealthy provegetarian food pattern.
*For the healthy and unhealthy provegetarian food pattern, canned fruit was excluded from the fruit item.
The provegetarian food pattern did not differentiate plants foods as healthy or unhealthy.
In the provegetarian food pattern, consumption of potatoes boiled/baked or chips/French fries and whole grains and refined grains were grouped as the "potatoes" and "cereal" grains food groups, respectively.

Table S2. Baseline characteristics of pre-pregnancy study population according to quintiles of the healthy provegetarian food pattern

	**Quintiles of the healthy provegetarian food pattern before pregnancy**				
	**Q1 31- 51**	**Q2 52- 55**	**Q3 56- 58**	**Q4 59- 63**	**Q5 64- 86**
Participants, n	765	784	619	829	592
Mean of the healthy provegetarian food pattern	47.9 (2.9)	53.6 (1.1)	57.0 (0.8)	60.8 (1.4)	67.9 (3.8)
Age at baseline (years)	26.9 (4.1)	27.5 (4.1)	27.6 (4.3)	28.2 (4.3)	28.3 (4.4)
BMI at baseline (kg/m^2)	21.2 (2.6)	21.3 (2.7)	21.3 (2.5)	21.5 (2.6)	21.3 (2.6)
Physical activity (METs/h/wk)	16.3 (19.2)	17.5 (18.8)	18.5 (18.8)	21.7 (22.1)	23.1 (21.6)
Parity (%)					
Nulliparous	85.0	83.4	81.9	82.3	82.3
1 pregnancy	7.2	7.9	9.0	9.0	8.6
2 pregnancies	3.5	3.4	3.6	3.7	4.2
≥3 pregnancies	2.4	2.4	3.1	2.5	1.2
Missing	2.0	2.8	2.4	2.4	3.7
Family history of diabetes mellitus (%)	8.5	10.2	10.8	11.3	10.1

Hypertension (%)	0.8	0.8	1.1	1.8	1.0
Smoking status (%)					
Never	59.3	56.8	55.9	56.2	56.2
Current	24.1	26.0	25.8	26.3	24.2
Former	16.6	17.2	18.3	17.5	19.6
Alcohol intake (g/day)	3.5 (4.7)	3.6 (4.9)	3.8 (4.8)	4.0 (6.1)	4.3 (5.0)
Pregnancies during Follow-up (%)					
1 pregnancy	46.4	44.0	46.4	48.9	55.4
2 pregnancies	31.1	36.1	33.9	32.7	27.7
≥3 pregnancies	22.5	19.9	19.7	18.5	16.9
Between-meal snacking (%)	51.2	44.8	39.6	38.8	36.3
Following special diets (%)	2.0	4.5	7.3	9.0	12.2
Television viewing (h/day)	1.7 (1.2)	1.6 (1.2)	1.7 (1.3)	1.6 (1.2)	1.5 (1.2)
Years of university education	4.7 (1.2)	4.7 (1.1)	4.7 (1.2)	4.7 (1.2)	4.7 (1.2)
Adherence to MedDiet (0-9 points)	2.9 (1.4)	3.4 (1.5)	3.9 (1.5)	4.5 (1.5)	5.4 (1.5)

Abbreviations: METs, metabolic equivalents; MedDiet, Mediterranean diet.

Table S3. Baseline characteristics of pre-pregnancy study population according to quintiles of the unhealthy provegetarian food pattern

	**Quintiles of the unhealthy provegetarian food pattern before pregnancy**				
	**Q1 36- 51**	**Q2 52- 55**	**Q3 56- 59**	**Q4 60- 63**	**Q5 64- 82**
Participants, n	817	765	756	603	648
Mean of the unhealthy provegetarian food pattern	47.8 (2.9)	53.5 (1.1)	57.5 (1.1)	61.4 (1.1)	68.1 (3.8)
Age at baseline (years)	28.2 (4.3)	28.0 (4.2)	27.7 (4.4)	27.3 (4.3)	26.9 (4.1)
BMI at baseline (kg/m^2)	21.7 (2.7)	21.3 (2.6)	21.4 (2.6)	21.1 (2.4)	21.1 (2.5)
Physical activity (METs/h/wk)	22.4 (23.5)	19.6 (19.5)	20.0 (20.4)	17.2 (18.3)	16.2 (17.8)
Parity (%)					
Nulliparous	82.3	82.1	82.7	85.2	83.5
1 pregnancy	8.4	9.2	8.3	6.8	8.6
2 pregnancies	3.7	4.7	3.3	3.5	3.1
≥3 pregnancies	2.7	2.2	2.2	1.8	2.6
Missing	2.9	1.8	3.4	2.7	2.2
Family history of diabetes mellitus (%)	11.8	9.2	9.9	10.4	9.6
Hypertension (%)	1.6	1.7	0.5	0.8	0.8
Smoking status (%)					

Never	54.0	55.0	56.7	62.5	58.0
Current	23.9	26.5	25.9	22.6	27.6
Former	22.2	18.4	17.3	14.9	14.4
Alcohol intake (g/day)	4.0 (5.2)	3.8 (4.6)	3.6 (4.7)	3.8 (5.0)	4.0 (6.5)
Pregnancies during Follow-up (%)					
1 pregnancy	51.7	49.5	48.4	43.6	44.8
2 pregnancies	30.1	30.7	34.0	35.7	32.9
≥3 pregnancies	18.2	19.7	17.6	20.7	22.4
Between-meal snacking(%)	34.8	39.0	41.3	43.6	56.8
Following special diets (%)	12.4	7.3	7.0	2.7	2.5
Television viewing (h/day)	1.5 (1.2)	1.5 (1.1)	1.7 (1.3)	1.7 (1.3)	1.8 (1.3)
Years of university education	4.8 (1.2)	4.7 (1.2)	4.7 (1.2)	4.7 (1.1)	4.7 (1.1)
Adherence to MedDiet (0-9 points)	4.9 (1.6)	4.1 (1.7)	3.7 (1.7)	3.6 (1.7)	3.4 (1.5)

Abbreviations: METs, metabolic equivalents; MedDiet, Mediterranean diet.

Table S4. Dietary intake of pre-pregnancy study population according to quintiles of the healthy provegetarian food pattern

	**Quintiles of the healthy provegetarian food pattern before pregnancy**				
	**Q1 31- 51**	**Q2 52- 55**	**Q3 56- 58**	**Q4 59- 63**	**Q5 64- 86**
Participants, n	765	784	619	829	592
Mean of the healthy provegetarian food pattern	47.9 (2.9)	53.6 (1.1)	57.0 (0.8)	60.8 (1.4)	67.9 (3.8)
Total energy intake (kcal/day)	2518 (719)	2394 (731)	2397 (721)	2465 (745)	2694 (734)
Carbohydrate (% of energy)	43 (7)	43 (7)	43 (7)	44 (7)	45 (8)
Protein (% of energy)	18 (3)	18 (3)	18 (3)	18 (3)	18 (3)
Fat (% of energy)	38 (6)	38 (6)	37 (6)	37 (7)	36 (7)
SFA (% of energy)	14 (3)	13 (3)	13 (3)	12 (3)	11 (3)
TFA (% of energy)	0.4 (0.2)	0.4 (0.2)	0.4 (0.2)	0.4 (0.2)	0.3 (0.2)
PUFA (% of energy)	6 (2)	5 (2)	5 (2)	5 (2)	5 (2)
MUFA (% of energy)	16 (3)	16 (4)	16 (4)	16 (4)	16 (4)
Total dietary fibre intake (g/day)	17 (7)	20 (8)	22 (8)	25 (9)	34 (13)
Sodium intake (mg/day)	4148 (2271)	3581 (2292)	3384 (2123)	3398 (3913)	3223 (1851)
Food group intake (g/day)					
Vegetables	375 (229)	473 (285)	559 (275)	642 (320)	835 (464)
Boiled/ baked potato	21 (22)	24 (27)	27 (28)	29 (34)	40 (38)
Potato chips/ French fries	39 (35)	30 (31)	26 (35)	23 (34)	17 (26)

Fruits*	182 (152)	255 (229)	308 (240)	364 (304)	532 (424)
Fruit juice	73 (89)	68 (99)	68 (93)	73 (116)	72 (106)
Legumes	18 (12)	20 (15)	22 (15)	24 (15)	29 (26)
Nuts	4 (5)	5 (7)	5 (7)	7 (12)	14 (19)
Refined grains	98 (62)	87 (67)	82 (58)	82 (64)	83 (68)
Whole grains	4 (16)	8 (21)	11 (23)	20 (41)	36 (48)
Olive oil	14 (11)	17 (14)	20 (15)	23 (16)	28 (17)
Animal fat	1.6 (2.8)	1.3 (2.8)	0.9 (2.1)	0.8 (2.2)	0.5 (1.9)
Dairy	512 (281)	476 (289)	466 (262)	464 (270)	460 (290)
Fish and seafood	94 (56)	95 (77)	94 (53)	103 (77)	109 (81)
Meat and meat products	207 (84)	190 (83)	185 (81)	175 (100)	166 (89)
Eggs	27 (15)	25 (20)	23 (15)	20 (12)	19 (16)
Sugar-sweetened beverages	104 (112)	89 (139)	68 (99)	63 (101)	47 (94)
Pastries	67 (54)	57 (52)	53 (53)	51 (49)	51 (57)
Coffee	42 (55)	52 (57)	55 (59)	64 (61)	75 (64)
Miscellaneous¶	34 (38)	26 (38)	23 (35)	22 (71)	15 (29)

Abbreviations: SFA, saturated fatty acid; TFA, trans fatty acid; PUFA, polyunsaturated fatty acid; MUFA, monounsaturated fatty acid.

*Fruits not included canned fruits. ¶ Miscellaneous: pizza, instant soups, mayonnaise.

Table S5. Dietary intake of pre-pregnancy study population according to quintiles of the unhealthy provegetarian food pattern

	**Quintiles of the unhealthy provegetarian food pattern before pregnancy**				
	**Q1**<br>**36- 51**	**Q2**<br>**52- 55**	**Q3**<br>**56- 59**	**Q4**<br>**60- 63**	**Q5**<br>**64- 82**
Participants, n	817	765	756	603	648
Mean of unhealthy provegetarian food pattern	47.8 (2.9)	53.5 (1.1)	57.5 (1.1)	61.4 (1.1)	68.1 (3.8)
Total energy intake (kcal/day)	2293 (653)	2258 (664)	2441 (703)	2596 (716)	2954 (751)
Carbohydrate (% of energy)	41 (7)	43 (7)	43 (7)	44 (7)	46 (7)
Protein (% of energy)	20 (3)	19 (3)	18 (3)	17 (2)	16 (2)
Fat (% of energy)	37 (7)	37 (7)	38 (7)	37 (7)	37 (6)
SFA (% of energy)	12 (3)	13 (3)	13 (3)	13 (3)	13 (4)
TFA (% of energy)	0.3 (0.2)	0.4 (0.2)	0.4 (0.2)	0.4 (0.2)	0.4 (0.2)
PUFA (% of energy)	5 (1)	5 (1)	5 (2)	5 (2)	6 (2)
MUFA (% of energy)	17 (4)	16 (4)	16 (4)	16 (4)	16 (3)
Total dietary fibre intake (g/day)	26 (10)	22 (10)	23 (11)	22 (10)	23 (10)
Sodium intake (mg/day)	3328 (2108)	3340 (2159)	3495 (2129)	3652 (2258)	4138 (4294)
Food group intake (g/day)					
Vegetables	727 (340)	589 (343)	551 (384)	483 (315)	430 (285)

Boiled/ baked potato	31 (30)	27 (29)	28 (35)	26 (31)	25 (28)
Potato chips/ French fries	14 (16)	19 (20)	26 (31)	34 (34)	52 (48)
Fruits*	386 (260)	307 (269)	309 (311)	313 (357)	269 (299)
Fruit juice	59 (85)	66 (91)	71 (100)	78 (104)	84 (125)
Legumes	25 (17)	23 (15)	22 (21)	21 (17)	20 (16)
Nuts	8 (12)	6 (12)	6 (11)	6 (11)	6 (10)
Refined grains	60 (44)	73 (49)	83 (58)	99 (62)	130 (83)
Whole grains	25 (38)	18 (34)	14 (35)	9 (24)	8 (28)
Olive oil	22 (14)	20 (15)	19 (15)	20 (17)	19 (16)
Animal fat	1.0 (2.3)	1.0 (2.4)	1.1 (2.5)	1.2 (2.9)	1.0 (2.2)
Dairy	498 (267)	459 (270)	475 (305)	478 (282)	470 (269)
Fish and seafood	119 (66)	99 (73)	101 (88)	90 (56)	79 (50)
Meat and meat products	183 (79)	176 (79)	188 (94)	188 (103)	195 (92)
Eggs	24 (15)	23 (17)	23 (14)	23 (19)	22 (14)
Sugar-sweetened beverages	40 (65)	58 (80)	69 (82)	93 (123)	135 (174)
Pastries	33 (26)	41 (31)	53 (42)	66 (51)	99 (79)
Coffee	72 (60)	60 (59)	54 (60)	50 (60)	45 (58)
Miscellaneous ¶	24 (35)	25 (36)	24 (36)	24 (35)	26 (78)

Abbreviations: SFA, saturated fatty acid; TFA, trans fatty acid; PUFA, polyunsaturated fatty acid; MUFA, monounsaturated fatty acid.

*Fruits not included canned fruits. ¶ Miscellaneous: pizza, instant soups, mayonnaise.

Table S6. ORs (95% CIs) for gestational diabetes mellitus according to quintiles of the healthy provegetarian food pattern before pregnancy

	**Quintiles of the healthy provegetarian food pattern before pregnancy**					
	**Q1**<br>**31- 51**	**Q2**<br>**52- 55**	**Q3**<br>**56- 58**	**Q4**<br>**59- 63**	**Q5**<br>**64- 86**	**P-trend**
Median score	49	54	57	61	67	
Nº cases/total	36/765	43/784	33/619	44/829	22/592	
Crude model	1.0 (Ref.)	1.18 (0.75- 1.85)	1.14 (0.70- 1.85)	1.14 (0.72- 1.78)	0.78 (0.45- 1.34)	0.424
Model 1	1.0 (Ref.)	1.17 (0.74- 1.86)	1.03 (0.62- 1.71)	1.05 (0.66- 1.67)	0.75 (0.43- 1.29)	0.270
Model 2	1.0 (Ref.)	1.17 (0.73- 1.87)	1.07 (0.65- 1.77)	0.96 (0.59- 1.56)	0.63 (0.35- 1.12)	0.075

Abbreviations: Ref, reference. Values are odds ratios (OR) and 95% confidence intervals. Crude model: Unadjusted model; Model 1: Model adjusted for maternal age at first pregnancy or GDM diagnosis at the cohort (continuous) and pre-pregnancy BMI (continuous); Model 2: Model 1 + time between recruitment and the first pregnancy (continuous), university education (years; 3-4, 5-6, >6), smoking status (never, former, and current), physical activity (metabolic equivalent h/wk; tertiles), family history of diabetes (yes, no), number of pregnancies during the follow-up (1, 2,≥3), parity (nulliparous, 1, 2, ≥3, missing), time spent watching TV (hours; tertiles), hypertension (yes, no), following an special diet at baseline (yes, no), snacking (yes, no), total energy intake (kcal/d; tertiles).

Table S7. ORs (95% CIs) for gestational diabetes mellitus according to quintiles of the unhealthy provegetarian food pattern before pregnancy

	**Quintiles of the unhealthy provegetarian food pattern before pregnancy**					
	**Q1**<br>**36- 51**	**Q2**<br>**52- 55**	**Q3**<br>**56- 59**	**Q4**<br>**60- 63**	**Q5**<br>**64- 82**	**P-trend**
Median score	48	53	57	61	67	
Nº cases/total	41/817	35/765	40/756	26/603	36/648	
Crude model	1.0 (Ref.)	0.91 (0.57- 1.44)	1.06 (0.68- 1.65)	0.85 (0.52- 1.41)	1.11 (0.70- 1.76)	0.724
Model 1	1.0 (Ref.)	0.98 (0.60- 1.58)	1.15 (0.73- 1.82)	0.93 (0.56- 1.57)	1.29 (0.80- 2.09)	0.355
Model 2	1.0 (Ref.)	1.00 (0.61- 1.64)	1.13 (0.70- 1.83)	0.86 (0.50- 1.49)	1.13 (0.67- 1.91)	0.789

Abbreviations: Ref, reference. Values are odds ratios (OR) and 95% confidence intervals. Crude model: Unadjusted model; Model 1: Model adjusted for maternal age at first pregnancy or GDM diagnosis at the cohort (continuous) and pre-pregnancy BMI (continuous); Model 2: Model 1 + time between recruitment and the first pregnancy (continuous), university education (years; 3-4, 5-6, >6), smoking status (never, former, and current), physical activity (metabolic equivalent h/wk; tertiles), family history of diabetes (yes, no), number of pregnancies during the follow-up (1, 2, ≥3), parity (nulliparous, 1, 2, ≥3, missing), time spent watching TV (hours; tertiles), hypertension (yes, no), following an special diet at baseline (yes, no), snacking (yes, no), total energy intake (kcal/d; tertiles).
